# Bioink Composition and Printing Parameters for 3D Modeling Neural Tissue

**DOI:** 10.3390/cells8080830

**Published:** 2019-08-05

**Authors:** Valentina Fantini, Matteo Bordoni, Franca Scocozza, Michele Conti, Eveljn Scarian, Stephana Carelli, Anna Maria Di Giulio, Stefania Marconi, Orietta Pansarasa, Ferdinando Auricchio, Cristina Cereda

**Affiliations:** 1 Department of Brain and Behavioural Sciences, University of Pavia, Via Forlanini 6, 27100 Pavia, Italy; 2 Laboratory of Neurobiology and Neurogenetic, Golgi-Cenci Foundation, Corso S. Martino 10, 20081 Abbiategrasso, Milan, Italy; 3 Genomic and post-Genomic Center, IRCCS Mondino Foundation, Via Mondino 2, 27100 Pavia, Italy; 4 Department of Civil Engineering and Architecture, University of Pavia, Via Ferrata 3, 27100 Pavia, Italy; 5 Department of Orthopaedics and Traumatology, Fondazione IRCCS Policlinico San Matteo, Str. Campeggi 18, 27100 Pavia, Italy; 6 Department of Biology and Biotechnology ‘L. Spallanzani’, University of Pavia, Via Ferrata 9, 27100 Pavia, Italy; 7 Laboratory of Pharmacology, Department of Health Sciences, University of Milan, Via A. di Rudinì 8, 20142 Milan, Italy; 8 Pediatric Research Center Fondazione Romeo ed Enrica Invernizzi, University of Milan, via Giovanni Battista Grassi 74, 20157 Milan, Italy

**Keywords:** disease modeling, 3D bioprinting, cell culture, neuroblastoma cell line, iPSC, neural stem cell, bioink, 3D cell culture, sodium alginate, gelatin

## Abstract

Neurodegenerative diseases (NDs) are a broad class of pathologies characterized by the progressive loss of neurons in the central nervous system. The main problem in the study of NDs is the lack of an adequate realistic experimental model to study the pathogenic mechanisms. Induced pluripotent stem cells (iPSCs) partially overcome the problem, with their capability to differentiate into almost every cell types; even so, these cells alone are not sufficient to unveil the mechanisms underlying NDs. 3D bioprinting allows to control the distribution of cells such as neurons, leading to the creation of a realistic in vitro model. In this work, we analyzed two biomaterials: sodium alginate and gelatin, and three different cell types: a neuroblastoma cell line (SH-SY5Y), iPSCs, and neural stem cells. All cells were encapsulated inside the bioink, printed and cultivated for at least seven days; they all presented good viability. We also evaluated the maintenance of the printed shape, opening the possibility to obtain a reliable in vitro neural tissue combining 3D bioprinting and iPSCs technology, optimizing the study of the degenerative processes that are still widely unknown.

## 1. Introduction

Neurodegenerative diseases (NDs) are a broad class of pathologies that lead to the death of a specific group of neural cells [1]. Despite the fact that some are quite widespread (such as Parkinson’s disease and Alzheimer’s disease), the exact cause of these disorders is not yet fully understood. The main limit of the study of NDs is the lack of a life-like model of the neural tissue, because of the difficult human reparability. Thus, it is necessary to develop a model system that can realistically recapitulate the pathogenetic mechanisms.

The employment of stem cells represents one of the most promising technology in the field of regenerative medicine and tissue engineering. They are used also for modeling human neurodegeneration, playing an important role in the study of these diseases [2,3,4,5]. Furthermore, with the discovery that adult somatic cells can be reprogrammed into induced pluripotent stem cells (iPSCs) it is now possible to generate stem cell lines with minimally invasive techniques, such as skin biopsies or, more recently, blood withdrawals [6]. The possibility to obtain iPSCs from patients and differentiate them into disease-specific cell types has led to an increased understanding of human NDs mechanisms and possible drug screening studies. However, the potential of iPSCs to recapitulate complex NDs remains incompletely exploited.

The development of 3D bioprinting provided an innovative tool to generate 3D cell cultures, creating “physiologic” models in which cells can be disposed in a controlled manner, growing in a tissue-like environment [7]. The creation of a 3D printed structure that can resemble the human neural tissues will allow the study of NDs using a “brain-in-a-dish” approach [8]. The combination of 3D bioprinting and iPSCs technology will allow the development of a more realistic and reliable in vitro cell culture, leading to the obtainment of neural organoids derived from patients’ cells, which will enable a personalized medicine approach in drug testing [8]. Until now, very few studies focused on the use of 3D bioprinting in the generation of neural tissue compared to other widely studied tissues, such as skin, bones, heart tissue, and cartilaginous structures [9]. These studies, which employed neural cells in 3D bioprinting process, presented some limitations [10,11], such as the poor characterization of bioinks that need to be fully developed and defined, because of the delicacy of the neural tissue [12].

Thus, the aim of this work was the development of a bioink that supports and helps the growth of iPSCs and their differentiation into neural stem cells (NSCs). We also evaluated the effect of the bioink on neural cell lines, providing the first step for the generation of a more complex and realistic neural tissue 3D model for the study of neurodegenerative diseases.

## 2. Materials and Methods

### 2.1. SA-GEL-Based Bioink

#### 2.1.1. Bioink Preparation

Bioink is composed of sodium alginate (SA) and gelatin (GEL) combined at different concentrations. Powder of SA (Sigma Aldrich, Milan, Italy) and GEL (Sigma Aldrich, Milan, Italy) was dissolved separately into sterile phosphate buffer saline (PBS) (Sigma Aldrich, Milan, Italy) and subsequently mixed at different concentrations (Table 1).

GEL was stirred at 72 °C using a magnetic plate and the SA solution was added to the GEL solution. All the steps were performed under sterile conditions. Bioinks were used immediately after preparation and sterilization. The bioink was printed using two controlled temperature, 25 °C and 37 °C, setting in each case the optimal printing pressure.

#### 2.1.2. Sterilization Process

Four sterilization methods were tested to obtain a sterile bioink: filtration, autoclaving, UV exposure, and pasteurization. The first method was filtration, based on the insertion of the SA and GEL components dissolved in sterile PBS in 20 mL syringes and then passed into a 0.20 μm filter (Corning, Corning, NY, USA). The second method was UV exposure, where the two powders, dissolved in sterile PBS, were placed under UV rays for 1 h. The third method tested was pasteurization where the SA and GEL solutions, placed in 50 mL centrifuge tube, were placed in a 72 °C bath for 1 h. The last method tested was autoclaving: the two components dissolved in sterile PBS were collected in 50 mL centrifuge tube and autoclaved at 121 °C for 20 min.

### 2.2. Bioplotter and Printing Set-Up

Cellink INKREDIBLE+ (Cellink AB, Gothenburg, Sweden) was used to print SA-GEL-based bioinks. The bioprinter was equipped with a chamber ventilated through High Efficiency Particulate Air (HEPA) filters, to guarantee a sterile environment during the printing process and UV system. The printhead mounted on our Cellink INKREDIBLE+ is not refrigerated. It can work at room temperature or at a higher temperature (25 °C and 37 °C, respectively). Cartridge was equipped with a 0.41 mm nozzle. The pressure, manually controlled, was changed according to the temperature and the composition of the bioink (Table 1). An open-source Computer-Aided Drafting (CAD) software was used to design STL grid model (FreeCAD©, ver. 0.16). The slicing process was assessed using Slic3r (version 1.2.9), the print-head speed was set at 600 mm/min and the layer height at 0.4 mm.

### 2.3. Repeatability Tests

The repeatability test consisted of three printed grids with 10 × 10 × 1.2 mm dimensions testing daily fresh bioink, and the tests were repeated 3 days consecutively. Each sample was imaged by a camera at a distance of 12 cm, obtaining a planar picture. The images were acquired and analyzed by ImageJ software (version 1.50i, NIH).

### 2.4. Printing of SH-SY5Y Cell Line

#### 2.4.1. SH-SY5Y Cell Line Cultures

The human neuroblastoma cell line SH-SY5Y was cultivated in DMEM supplemented with 15% fetal bovine serum (FBS), penicillin/streptomycin (100 U/mL; 100 mg/mL), and 2 mM L-glutamine, at 37 °C in a humidified atmosphere containing 5% CO_2_.

#### 2.4.2. Encapsulation of SH-SY5Y Cell Lines

Sterilized bioink and cells were transferred in a 5 mL syringe. The “luer” connector was used to improve cell mixing and to encapsulate cells in the bioink. Cells were cultivated for 5 days in a 12-well plate and the medium was changed daily. Hereinafter, cell-laden bioink after printing process will be indicated as 3D printed structure.

#### 2.4.3. Crosslinking Process

The crosslinking process was based on bivalent ions of calcium chloride (CaCl_2_) that led to reticulate SA residue. Crosslinking was obtained by diluting CaCl_2_ powder (Sigma Aldrich, Italy) in sterile distilled water at a final concentration of 2% and maintained for 5 min on the 3D printed structure. After this, CaCl_2_ was removed and 3D printed structures were washed in sterile PBS.

### 2.5. Cell Viability Assay on Crosslinked Structures

The viability test for 3D printed structures was performed with LIVE/DEAD Cell Viability Assay (Invitrogen, Carlsbad, CA, USA) following manufacturer instructions. It is based on two fluorescent dyes, calcein AM (green) for living cells and ethidium homodimer-1 (red) for dead cells. For the viability assay, SH-SY5Y cells were suspended to a final concentration of 5 × 10^4^ cells/mL. After the stain with calcein AM and ethidium homodimer-1, 3 fields for each sample were manually counted. The correction of pre-print viability was used to calculate the exact percentage of living cells after the printing process:

(1) $$Cell\;viability=\frac{\mathrm{cell}\;\mathrm{viability}\;\left(\%\right)}{\mathrm{pre}-\mathrm{print}\;\mathrm{cell}\;\mathrm{viability}\;\left(\%\right)}*100$$

### 2.6. Immunofluorescence Analysis

3D printed structures were washed and fixed with 4% paraformaldehyde (PFA) (Sigma Aldrich, Italy) and subsequently rinsed with ice-cold PBS. The 3D printed structures were incubated at RT with blocking solution (10% normal goat serum and 0.3% Triton X100 in PBS). Incubation was performed in blocking solution overnight (ON) at 4 °C with the following primary antibodies: mouse monoclonal anti-GAPDH (GeneTex, Irvine, CA, USA; dilution 1:200) and anti-α-Tubulin (Cell Signalling Technology, The Netherlands; dilution 1:200). The fluorescent-tagged secondary antibody CF 488A donkey anti-mouse (Sigma-Aldrich; dilution 1:700) was used for detection. Slides were mounted with Prolong^®^ Gold anti-fade reagent with 4′6-diamidino-2-phenylindole (DAPI; Invitrogen) and images were acquired by Axio Imager 2 fluorescence microscope (Zeiss, San Diego, CA, USA) equipped with Axiocam Mrm camera and Leica SP8 microscope confocal system.

### 2.7. Generation of iPSCs from Healthy Volunteers and Cellular Differentiation

#### 2.7.1. Healthy Volunteers’ Enrollment

Healthy volunteers were recruited at the IRCCS Policlinico S. Matteo Foundation in Pavia. Healthy volunteers were all unrelated and the normal phenotype was confirmed by interviews based on personal health histories. The study design was examined by theInstitutional Review Board (IRBs) of the enrolling institutions. All individuals joined in the study signing the consensus after reading informative note (see Section 2.10).

#### 2.7.2. Isolation of PBMCs

Peripheral blood mononuclear cells (PBMCs) were immediately isolated from blood using Histopaque^®^-1077 (Sigma-Aldrich, Italy) following manufacturer’s instructions. Cells viability was assessed by trypan blue (Sigma Aldrich, Italy) exclusion test with an automatic cells counter, and cells were cryopreserved in FBS and dimethyl sulfoxide (DMSO) (Sigma Aldrich, Italy) to prevent cells death. PBMCs were placed at −80° C into a container, which provides a rate of cooling close to −1 °C/min. Finally, cells were stored into a cryogenic storage tank with liquid nitrogen.

#### 2.7.3. PBMCs Reprogramming

**Figure 1:**
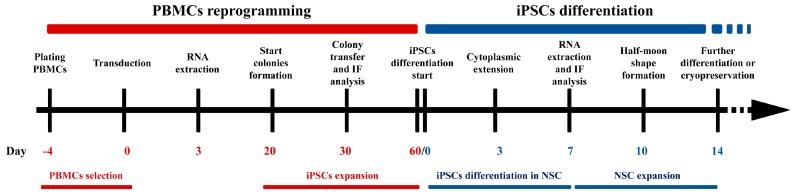
Timetable of the peripheral blood mononuclear cells (PBMCs) reprogramming protocol (in red) and of induced pluripotent stem cell (iPSC) differentiation in neural stem cells (NSCs) (in blue), with the characteristic checkpoints used in the protocol.

5 × 10^5^ PBMCs were resuspended in 24-well plates with PBMCs medium (StemPro™-34 + SCF 100 ng/mL, FLT-3 100 ng/mL, IL-3 20 ng/mL, IL-6 20 ng/mL). Viruses provided by CytoTune^®^-iPS 2.0 Sendai Reprogramming Kit (Invitrogen, USA) were incubated for 24 h. The medium was replaced with fresh complete PBMCs medium to remove the CytoTune™ 2.0 Sendai reprogramming vectors. After 2 days, transduced cells were plated on vitronectin-coated culture dishes in complete StemPro™-34 medium (Invitrogen, USA) and the consumed medium was replaced every day for 4 days. After 7 days from transduction, cells were grown in Essential 8 Medium (Thermo Fisher Scientific, Waltham, MA USA). Consumed medium was replaced every two days for 3 weeks. Undifferentiated colonies were manually picked and plated on vitronectin-coated well. Colonies were split using 0.5 mM EDTA for 3–5 passages. Finally, cells were collected using 0.5 mM EDTA (Thermo Fisher Scientific) and cryopreserved in liquid nitrogen in Essential 8 Medium plus 10% DMSO (Figure 1).

#### 2.7.4. Neural Stem Cells Differentiation from iPSCs

The differentiation protocol of iPSCs into NSC is based on PSC Neural Induction Medium (Invitrogen, USA). iPSCs at 5th passage were seeded in 6-well plate and coated with vitronectin. When cells reached the 60–70% confluency, the medium was changed from Essential 8 to Neurobasal (Invitrogen, USA) supplemented with Neural Induction Supplement 50× (Invitrogen, USA) for 7 days, refreshing the medium daily. After 7 days, the iPSCs differentiated into NSCs. Cells were then split using Stem Pro Accutase to expand cells and cryopreserved for further differentiation protocols (Figure 1).

Efficient reprogramming was assessed by immunofluorescence analysis of stemness markers, using Pluripotent Stem Cell 4-Marker Immunocytochemistry Kit (Invitrogen, USA), containing anti-SSEA4, anti-OCT4, anti-SOX2, and anti-TRA 1-60, following manufacturer’s instructions. Cells were incubated with 4% PFA at RT and permeabilized with saponin 1%. After incubation with blocking solution, primary antibodies were added and cells were incubated ON at 4 °C. Cells were rinsed with wash buffer and secondary antibodies were added at RT. Samples were washed, mounted with NucBlue^®^ Fixed Cell Stain (DAPI) (Invitrogen, USA), dried, nail-polished, and analyzed by confocal microscopy. The same protocol was used for NSCs using Human Neural Stem Cell Immunocytochemistry Kit (Invitrogen, USA). To assess the neural stemness of the cells, the neuronal markers Nestin, SOX2, SOX1, and PAX6 were detected.

#### 2.7.5. PCR and RT-qPCR

Total RNA from iPSCs and NSCs cells was extracted using TRIzol^®^ (Invitrogen, Italy) following manufacturer’s instructions. RNA quality and quantity were determined with the NanoDrop spectrophotometer (Invitrogen, USA) and 1 μg was reverse-transcribed using the iScriptcDNA Synthesis Kit (BioRad, Italy) following the manufacturer’s protocol. PCR amplifications were performed with 10 μL of cDNA, using MyTaq (Bioline, UK), and the following amplification protocol: denaturation at 95 °C for 30 s, annealing at 55 °C for 30 s, and elongation at 72 °C for 30 s, for 33 cycles. Products were analyzed using 2% agarose gel electrophoresis. Primer sequences are listed in Table 2.

PCR amplifications were performed with the CFX Connect™ Real-Time PCR Detection System (BioRad, Italy) using SYBR Green Master Mix (BioRad, Italy). The GAPDH gene was used as housekeeping gene to normalize values. Primer sequences and annealing temperature are listed in Table 3.

We normalized the expression of the four genes using the housekeeping gene GAPDH.

### 2.8. Encapsulation of iPSCs and NSCs and Relative Cell Viability Assay

The encapsulation of iPSCs and NSCs was assessed as previously described (see Section 2.4.2). For the relative cell viability assay iPSCs and NSCs cells were resuspended at a concentration of 2 × 10^6^ cells/mL each. Cells were cultivated for 7 days in a 12-well plate and the medium was changed daily. Day 0 was set at 100%, and the viability at day 3 and day 7 was calculated using day 0 as the control.

### 2.9. Statistical Analysis

The tests were performed three times or more. Each parameter and result are shown as means ± standard deviation (SD). We performed statistical analysis using GraphPad Prism (version 7, GraphPad Software), adopting the one-way analysis of variance test (ANOVA), followed by Newman-Keuls Multiple Comparison Test. Values were considered statistically significant when *p* -values were <0.05.

### 2.10. Ethic STATEMENT

All procedures performed in studies involving human participants were in accordance with the ethical standards of the institutional and/or national research committee and with the Helsinki declaration and its later amendments or comparable ethical standards. The study design was examined by the IRBs of the enrolling institutions (Protocol n°375/04–version 07/01/2004).

## 3. Results

### 3.1. Quality Settings of SA-GEL-Based Bioink with Cellink INKREDIBLE+ Bioplotter

The choice of the optimal mix of materials suitable for 3D cell culture was the first step to create a good bioink with well-defined characteristics.

SA and GEL were previously described for their high biocompatibility with many cell lines and tissues such as skin, muscle, and liver [13,14] and for these reasons they were tested. Two compositions of bioinks, 4% SA and 4% GEL and 6% SA and 4% GEL, with two different printing temperatures, i.e., room temperature (25 °C) and physiological temperature (37 °C), were used (Table 1). The best printing pressure was also set and measured (Table 1).

Pressure range needed to print 6% SA and 4% GEL at 25 °C (sample 1, Table 1) was higher because of a highest viscosity of the bioink, compared to the same bioink (6% SA and 4% GEL) printed at 37 °C (sample 2, Table 1). We also tested the printing pressure using a bioink composed of 4% SA and 4% GEL, and we obtained a higher printing pressure at 25 °C compared with the same bioink, printed at 37 °C (sample 3 and 4 each, Table 1). A difference in printing pressure was appreciable between sample 1 (6% SA and 4% GEL) and sample 3 (4% SA and 4% GEL), indicating a major viscosity of sample 1 probably due to a high concentration of SA (6% vs. 4%). Other concentrations were previously tested for cellular viability and compared with 2D control (see Figure S1). The good viability was obtained with 4% SA and 4% GEL.

**Figure 2:**
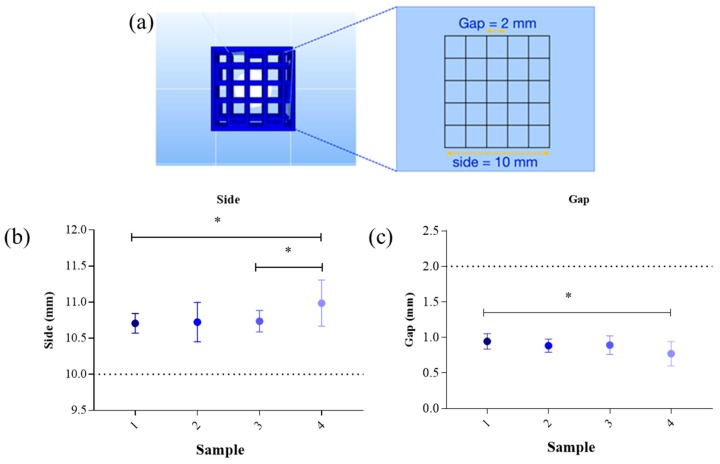
Repeatability tests using samples listed in Table 1: (**a**) CAD image of a grid with gap dimension and side dimension; (**b**) Dimension of side of samples shown in Table 1. Both sample 1 and sample 3 show a statistically significant difference compared to sample 4 (* *p* < 0.05). Dotted line indicates the reference value; (**c**) Dimension of gap of samples shown in Table 1. Sample 1 shows a statistically significant difference compared to sample 4 (* *p* < 0.05). Dotted line indicates the reference value. Error bars indicate SD. Data were analyzed by ANOVA (n = 9), followed by Newman-Keuls Multiple Comparison Test; * *p* < 0.05.

With regards to the repeatability of the printed grid, the difference between the preestablished measures (dotted line in Figure 2b,c) and the effectiveness measures of printed grid were evaluated. The results of the side and gap measures were described in Figure 2.

The side dimension of samples printed at 37 °C (Figure 2b, sample 2 and 4) showed higher SD highlighting difficulties in maintaining the same dimension during the repeatability tests. This difference is related to the use of GEL, a temperature-dependent biomaterial, which liquefies with increasing temperatures. However, GEL has the advantage to mimic the extracellular matrices.

Side dimension showed a statistically significant difference between sample 1 and sample 4 due to a difference in the printing temperature and SA concentration. Sample 1 shows a better accuracy during the printing process with respect to the reference value (dotted line in Figure 2). Side dimension showed a statistically significant increase in sample 4 compared to sample 3, due to a different printing temperature (*p < 0.05, Figure 2b). Gap showed a statistically significant difference between sample 1 and sample 4, indicating the accuracy with respect to the reference value (*p < 0.05, sample 1 vs. 4, Figure 2b,c).

According to these results, the optimal bioink that maintains the preestablished dimension during repeatability tests was sample 1, composed of 6% SA and 4% GEL and printed at 25 °C. We also tested more complex structures, using 6% SA and 4% GEL (Figure S2), to ensure that our hydrogel will be suitable for further experiments that need different shape. We printed a multilayer solid cube (10 × 10 × 10 layer) and an empty cylinder (25 layer).

### 3.2. 6% SA and 4% GEL Showed a Good Viability with SH-SY5Y Cell Line

After the quality settings of SA-GEL-based bioink, the biocompatibility of the optimal bioink was tested using both concentrations of SA (6% and 4%). The custom bioink was sterilized by pasteurization to avoid contamination during the cell culture.

The sterile bioink was then mixed with SH-SY5Y cell suspension, to have 5 × 10^4^ cells/mL of bioink (i.e., 1.5 × 10^5^ cells in 3 mL of bioink). This cell-laden bioink was loaded in a 3 mL cartridge and printed using a temperature of 25 °C and a pressure of 55 kPa for 6%SA–4% GEL bioink and 45 kPa for 4% SA–4% GEL bioink. We decided to print 10 × 10 × 1.2 mm grid, 3 layers of 0.4 mm each.

**Figure 3:**
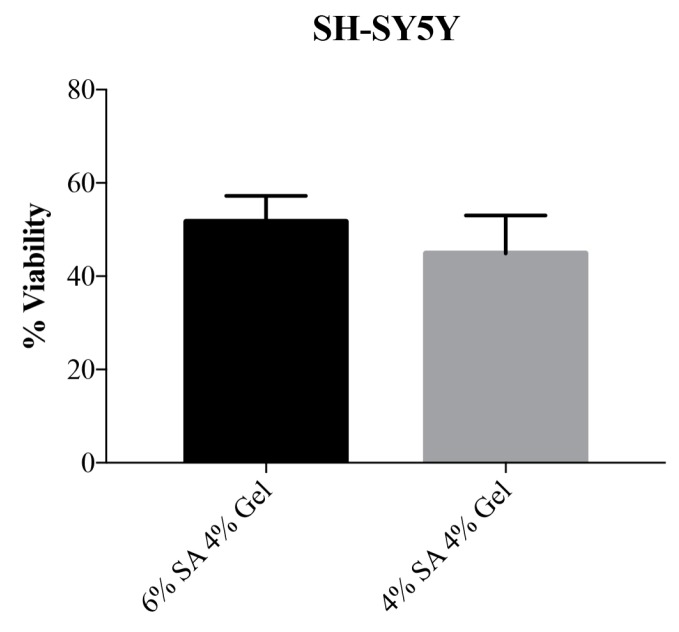
Viability test for SH-SY5Y cell line after 5 days of culture in the bioink. On the *y*-axis, the viability of cells is expressed as a percentage. Error bars indicate SD.

Viability results were obtained with Live/Dead cell viability assay (Invitrogen, USA). Three measures were taken from the same 3D printed structure and results were plotted as mean percentage of viability ± SD. In Figure S3 we reported a representative image of cells for the determination of cell viability. The obtained results confirmed the good cellular viability of SH-SY5Y cells in both tested bioink composition and no statistically significant differences were observed (Figure 3).

### 3.3. 3D printed Structures Maintained a Good Structure and a Defined Spatial Organization

After the confirmation of a good viability of SH-SY5Y encapsulated into the bioink, we have to confirm that the 3D printed structure maintains the shape after printing and crosslink, in particular, because of the presence of GEL in bioink the structure has to be stiffened to guarantee the integrity during culture in CO_2_ incubator at 37 °C.

**Figure 4:**
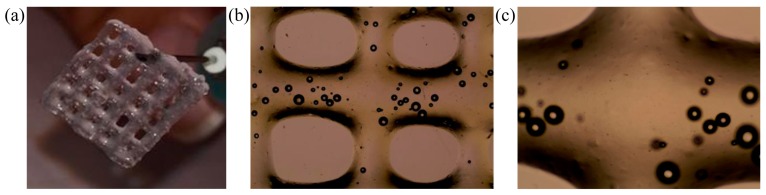
Crosslinked structure printed with Cellink INKREDIBLE+ (**a**) Crosslinked grid after 5 min in a bath with 2% CaCl_2_ (diluted in distilled water); (**b**) Phase-contrast microscope image captured at 4× (EVOS XL Core Cell Imaging System, Thermo Fisher, USA) of a printed grid with Cellink INKREDIBLE+ bioplotter. The presence of bubbles in the bioink does not interfere with the stability of the 3D printed structures; (**c**) 10× magnification of phase-contrast image.

Figure 4a shows a printed grid using Cellink INKREDIBLE+ after the crosslink with CaCl_2_. The grid resulted resistant and easy to handle, thus useful for cell cultures at 37 °C. Figure 4b,c shows a phase-contrast microscope image (4× and 10× magnification each) reporting the high definition of the printing process.

**Figure 5:**
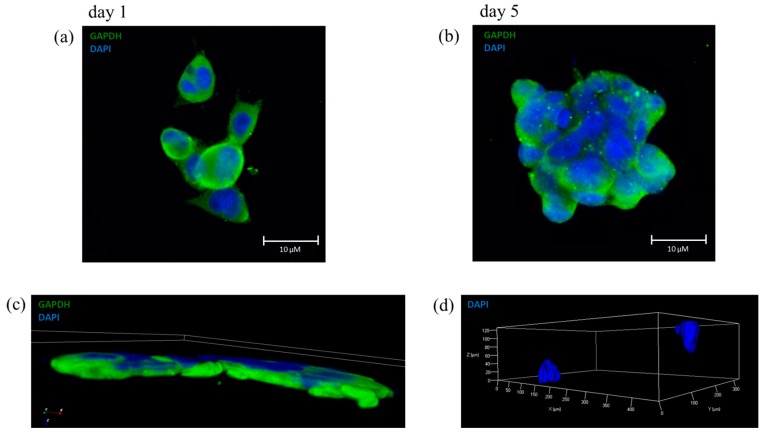
Immunofluorescence images of SH-SY5Y in 6% SA and 4% GEL. (**a**) Image of SH-SY5Y after 1 day of culture in 6% SA and 4% GEL, indicating the distribution of single cells. (**b**) The image shows the presence of several nuclei, indicating a good proliferation and a characteristically three-dimensional organization; GAPDH (green) labels cells and DAPI (blue) labels the nuclei; (**b**) Example of 3D reconstruction using ImageJ software (version 1.50i, NIH) of the confocal image: GAPDH (green) labels cells and DAPI (blue) labels the nuclei at day 5 of culture; (**c**–**d**) Reconstruction in three dimensions of a Z-stack obtained from Axio Imager 2 (Axiocam Mrm) in which cells were labeled with DAPI. The image shows the formation of two aggregation clusters to indicate cellular proliferation within 5 days of culture within bioink.

To be suitable for 3D bioprinting, our optimal bioink has to maintain a 3D localization of the included cells and this property was confirmed by immunofluorescence analysis (Figure 5).

Figure 5a shows cells encapsulated the day after the printing process, where only some cells overlap each other, and the shape of each single cell is clearly distinguishable. Figure 5b shows a clump with a large number of cells, indicating a good proliferation during 5 days of culture with no signs of cells migration, in comparison with Figure 5a. Figure 5c shows a 3D confocal reconstruction of a group of cells of the same immunofluorescence staining, where it is appreciable the three-dimensional proliferation of the cells within the bioink. Figure 5d shows a reconstruction in 3D of z-stack in which two distinct colonies, labeled with DAPI, can be observed. These two colonies were organized spatially in a 3D environment; they maintained their position during the 5 days of proliferation confirming the 3D structure of the optimal bioink. Moreover, we tested another shape to maintain the spatial organization by printing parallel lines with and without cells (Figure S4).

### 3.4. PBMCs-Derived iPSCs Remain Viable after 3D Bioprinting

The development of a patient-specific in vitro model suitable for disease modeling is and will be the main goal in the field of NDs. Recently, the use of PBMCs to reprogram iPSCs became fundamental to obtain specific neurons involved in many pathologies, where it is very difficult to obtain primary neurons derived from biopsy or autopsy material.

**Figure 6:**
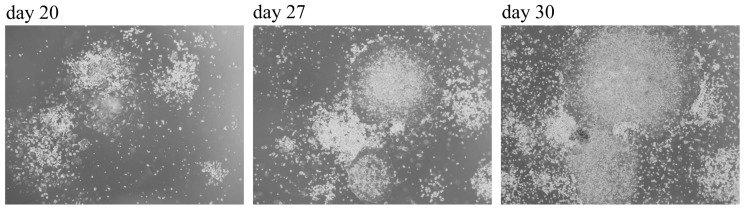
Phase-contrast image of PBMCs-derived iPSCs colonies after day 20, 27, and 30 after transduction. At day 20, cells started to grow adherent to vitronectin-coated plates, and organized themselves in colony, visible already at day 27. At day 30, cell reprogramming is evident: cells are organized in a well-defined perimeter lane, and they are concentrated into colonies. Cells that were not de-differentiated died because of the presence of a selective medium for iPSCs.

After two to three weeks since transduction with Yamanaka factors using the Sendai virus vector, cells became organized into a colony (Figure 6).

In Figure 6, cell colony grows in a round-shape with a well-defined perimeter lane.

**Figure 7:**
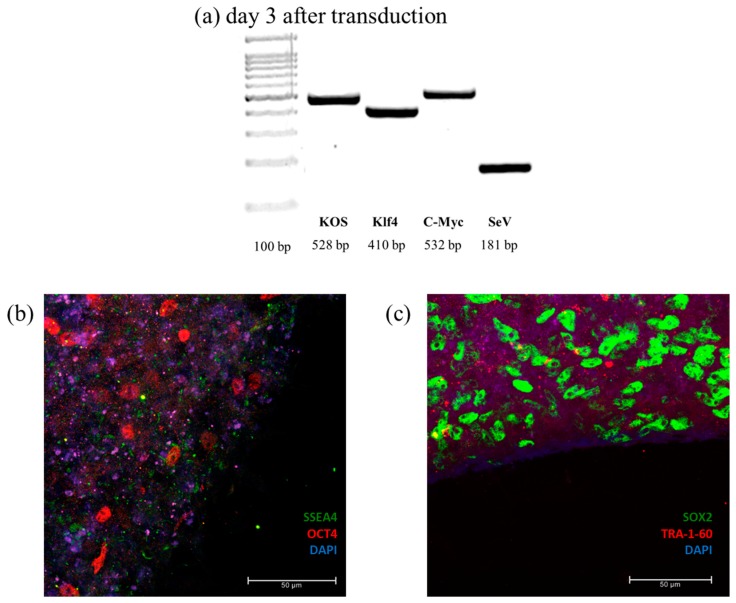
Characterization of 2D PBMCs reprogramming into iPSCs. (**a**) PCR of the viral RNA (SeV) and the three transgenes used during transduction: KOS, Klf4, and c-Myc. Sample at day 3 after transduction express all the transgenes, confirming the success of the transfection. (**b**,**c**) Immunofluorescence images of PBMCs-derived iPSCs using four stemness marker: SSEA4 and OCT4 (**b**), SOX2 and TRA1-60 (**c**), and DAPI (blue) labels the nuclei.

To confirm cellular reprogramming, PCR was applied to detect the presence of transgenic genes and of the vector used during transduction. Pluripotency was confirmed by verifying the expression of four pluripotency marker: OCT4, SOX2, SSEA4, and TRA-1-60 (Figure 7).

**Figure 8:**
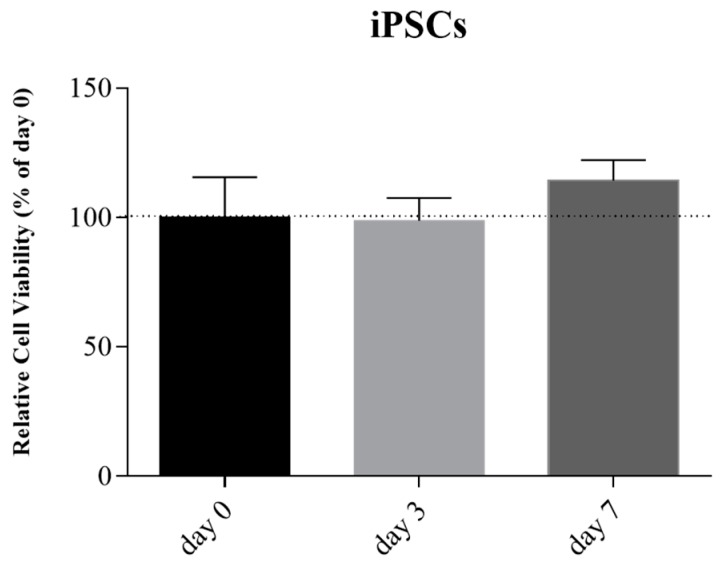
Relative cell viability of iPSCs encapsulated into 6% SA and 4% GEL bioink, at day 0, 3, and 7. The relative cell viability was expressed in percentage of day 0 (y-axis). Error bars indicate SD.

After the confirmation of pluripotency, the sterile bioink was mixed with iPSCs suspension and printed. The Live/Dead cell viability assay at day 0, 3, and 7 confirmed the maintenance of iPSCs cellular viability compared to day 0 and no statistical difference was observed (Figure 8).

### 3.5. Differentiated Neural Stem Cells from iPSCs Maintain a Good Viability in SA-GEL-Based Bioink

**Figure 9:**
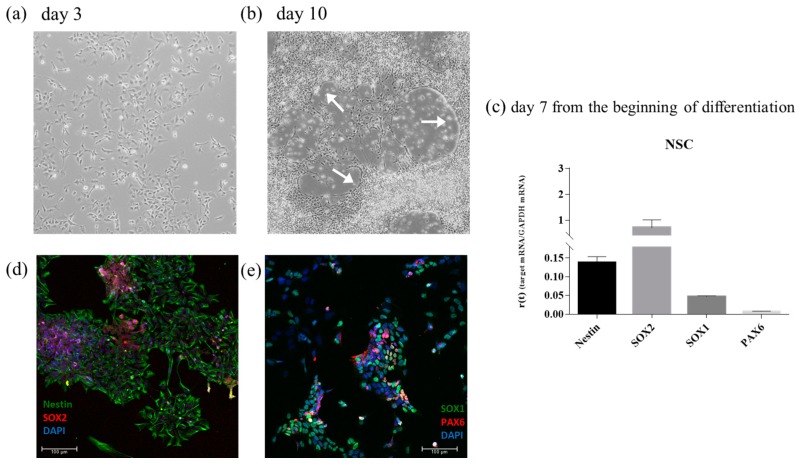
Characterization of iPSCs-derived NSC. (**a**) Phase-contrast image of NSC at day 3 of differentiation: cells show little cytoplasmic extension; (**b**) Phase-contrast image of NSC at day 10 of differentiation: cells were organized like half-moon shape (indicated with white arrow; (**c**) RT-qPCR of four genes: Nestin, SOX2, SOX1, and PAX6 conducted at day 7 since the beginning of the differentiation to neural stem cells; (**d**,**e**) Immunofluorescence images of iPSCs-derived NSC using four differentiation marker: Nestin and SOX2 (**d**), SOX1 and PAX6 (**e**), and DAPI (blue) labels the nuclei. Error bars indicate SD.

iPSCs were then differentiated into NSC to create a neural tissue like substrate. A standard protocol based on a single commercial medium was employed. At day 3 of differentiation, cells showed little cytoplasmic extension (Figure 9a). At day 10 of differentiation, cells were organized in a half-moon shape (indicated with a white arrow in Figure 9b) and the cytoplasmic extension was enhanced.

To further characterize the differentiation of NSC, RT-qPCR was used to detect the expression of four genes: Nestin, SOX2, SOX1, and PAX6 (Figure 9c). The corresponding proteins were also detected using immunofluorescence (Figure 9d,e).

NSCs were tested into our bioink, after the confirmation of the neural stemness state. The sterile bioink was mixed with NSCs suspension to reach 2 × 10^6^ cells/mL of bioink. NSCs encapsulated were printed and encapsulated into the bioink as described for iPSCs.

**Figure 10:**
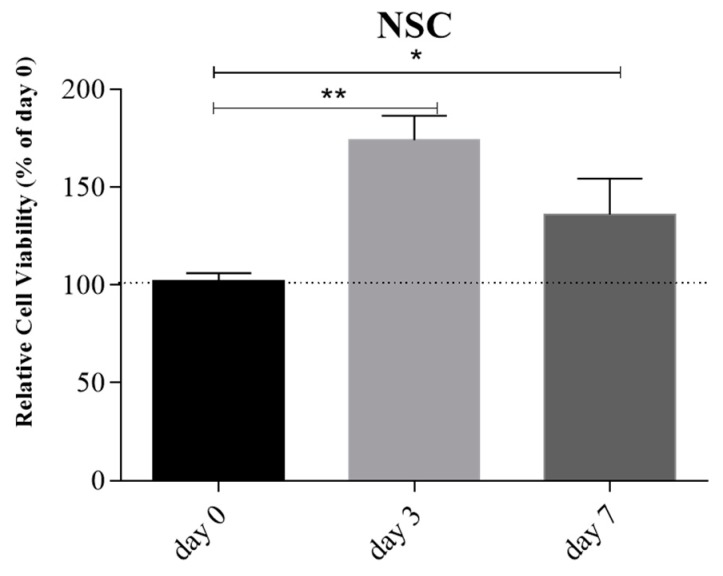
Relative cellular viability of NSCs encapsulated into 6% SA and 4% GEL bioink at day 0, 3, and 7. The relative cell viability is expressed as percentage (y-axis) of day 0. Both at day 3 and day 7, relative cell viability shows a statistically significant increase compared to day 0 (** *p* < 0.01; * *p* < 0.05, respectively). Error bars indicate SD. Data were analyzed by ANOVA (n = 3), followed by Newman–Keuls multiple comparison test; * *p* < 0.05 and ** *p* < 0.01.

Live/Dead cell viability assay at day 0, 3, and 7 confirmed the good cellular viability compared to day 0 during 7 days of culture (Figure 10).

## 4. Discussion

To this day, the lack of in vitro models for the study of degenerative pathologies is a central problem for researchers. Animal models are the most used models to address several scientific questions, from basic research, to identify disease mechanisms, to the development of novel therapies. Even so, the results obtained on animals are not always confirmed in humans [15]. Human *post-mortem* tissue also provides important information on disease mechanisms. However, this tissue has limited availability and does not allow considerations on disease progression [16]. Thus, in vitro models, in particular patient-derived iPSCs, can be used alongside with both animal models and post-mortem material to investigate brain disorders. These models are almost inexpensive research tools and allow the study of disease progression, thus allowing the understanding of disease mechanisms and the identification of novel therapeutic targets [17].

Moreover, in recent years, the transition from 2D to 3D culture systems has emerged and, through the incorporation of multiple cell types, 3D systems better simulate the in vivo milieu.

The combination of these two new technologies, i.e., iPSCs and 3D bioprinting, is recently used to model several diseases, reducing the number of animals used, the cost, and the duration of experiments. The possibility to obtain the desired cell type from somatic cells is crucial when it is difficult to obtain target tissues both to study and to treat. Moreover, 3D bioprinting adds the third dimension to the in vitro cell culture, generating a more reliable model for different tissues, such as bone, heart, and cartilage [8,18,19].

The present study describes the combination of iPSCs and 3D bioprinting technologies to model a neural tissue. At present, studies that combine neural cells and 3D bioprinting are poor and present as a major limitation of the variable and not standardized bioink composition. We identified sodium alginate (SA) and gelatin (GEL) as biomaterials at a concentration of 6% and 4%, respectively [13,14]. Thus, we tested the printability of our bioink with a Cellink INKREDIBLE+ bioplotter, and the printing process was accurately standardized regarding pressure, temperature, and velocity to print bioink composed of SA and GEL. We identified an optimal bioink composed of 6% SA and 4% GEL, printed at 25 °C to expand the cell line with a neuronal phenotype. Between the conditions of printing, the temperature is a fundamental parameter to consider for future test with cells encapsulated into the bioink because the excessive decrease or increase in temperature induces suffering in the cells and high mortality during printing. Furthermore, we established a good compromise between the capabilities of the bioink to maintain the desired shape after printing and the high viability and proliferation of cells encapsulated into the bioink.

Considering that the main focus of our work is the modeling of the neural tissue, we started by printing the SH-SY5Y cell line, a neuroblastoma cell line with neuronal phenotype. Our results demonstrated that the optimal conditions were 6% SA and 4% GEL, printed at 25 °C and with a pressure of 45–70 kPa. Under these conditions, cells displayed good viability after 5 days of culture. We also appreciated a three-dimensional arrangement of the proliferation of the cells within the bioink. Furthermore, cells organized themselves in distinct colonies in the 3D environment; they also maintained their position all along the 5 days of proliferation, confirming that the 3D structure was optimal for the proposed bioink.

To create an in vitro model for future tailored therapy, we used PBMCs-derived iPSCs and iPSCs-derived NSCs. Results using these cells further confirmed that our bioink is suitable for 3D bioprinting. PBMCs-derived iPSCs and iPSCs-derived NSCs maintained a good cellular viability for 7 days and a good spatial organization, fundamental for cell-to-cell communication and physiologically relevant for subsequent mechanistic studies.

The weakness of the neural cells and the lack of direct applicability in clinical therapies delayed, until now, the creation of an optimized and standardized bioink. Thus, in this work we set, for the first time, a protocol suitable for neuronal cultures. At present Matrigel-based cell cultures do not guarantee a defined 3D spatial assembling, indeed with this protocol, we established the repeatability of the 3D bioprinting protocol, which allows to control cells and differentiation factors deposition, and which facilitate cell spatial organization.

## Figures and Tables

**Table 1:** List of the characteristic of four different bioink batches.

Sample	SA	GEL	Printing Temperature ^1^	Printing Pressure (kPa) ^2^
1	6%	4%	*25 °C*	*45–70*
2	6%	4%	*37 °C*	*30–50*
3	4%	4%	*25 °C*	*35–60*
4	4%	4%	*37 °C*	*30–40*

^1^ Printing Temperature (°C): 25 °C and 37 °C made by temperature-controlled print head. ^2^ Printing Pressure (kPa): pressure used for an optimal bioink extrusion, setting by a pressure regulator.

**Table 2:** List of primers sequences used for PCR to detect transgene used during reprogramming protocol.

Target	Primer sequence (5′-3′)	Product Size
SeV	**F** GGA TCA CTA GGT GAT ATC GAG C **R** ACC AGA CAA GAG TTT AAG AGA TAT GTA TC	181 bp
Klf4	**F** TTC CTG CAT GCC AGA GGA GCC C **R** AAT GTA TCG AAG GTG CTC AA	410 bp
c-Myc	**F** TAA CTG ACT AGC AGG CTT GTC G **R** TCC ACA TAC AGT CCT GGA TGA TGA TG	532 bp
KOS	**F** ATG CAC CGC TAC GAC GTG AGC GC **R** ACC TTG ACA ATC CTG ATG TGG	528 bp

**Table 3:** List of primers sequences used for RT-qPCR and relative annealing temperature to detect the expression of stemness genes.

Gene	Primer sequence (5′-3′)	Annealing
Nestin	**F** GGA AGA GAA CCT GGG AAA GG **R** GAT TCA GCT CTG CCT CAT CC	60 °C
SOX2	**F** AGTCTCCAAGCGACGAAAAA **R** TTTCACGTTTGCAACTGTCC	60 °C
SOX1	**F** AAATACTGGAGACGAACGCC **R** AACCCAAGTCTGGTGTCAGC	60 °C
PAX6	**F** TGTGTGCTCTGAAGGTCAGG **R** CTGGAGCTCTGTTTGGAAGG	60 °C
GAPDH	**F** CAG CAA GAG CAC AAG AGG AAG **R** CAA CTG TGA GGA GGG GAG ATT	60 °C

## Supplementary Materials

The following are available online at https://www.mdpi.com/2073-4409/8/8/830/s1, Figure S1: Viability test (LIVE/DEAD^®^ Cell Viability Assays, Invitrogen, USA) for SH-SY5Y cell line after 5 days of culture in the bioink, Figure S2: Example of two complex structures: CAD and printed sample using 6% SA and 4% GEL, Figure S3: Cellular viability with the LIVE/DEAD^®^ cell viability assay (Invitrogen, USA) in SH-SY5Y encapsulated in 6% SA and 4% GEL after 5 days of culture. Figure S4: Representation of CAD image and fluorescence image of SH-SY5Y after 5 days of culture.
