# Supplementary material for: Bioink Composition and Printing Parameters for 3D Modeling Neural Tissue
Source: Cells. 2019 Aug 5;8(8):830. doi: 10.3390/cells8080830 (PMC6721723; doi:10.3390/cells8080830)
Supplement: Supplementary file 1 [file cells-08-00830-s001.pdf]

SUPPLEMANTARY FIGURES

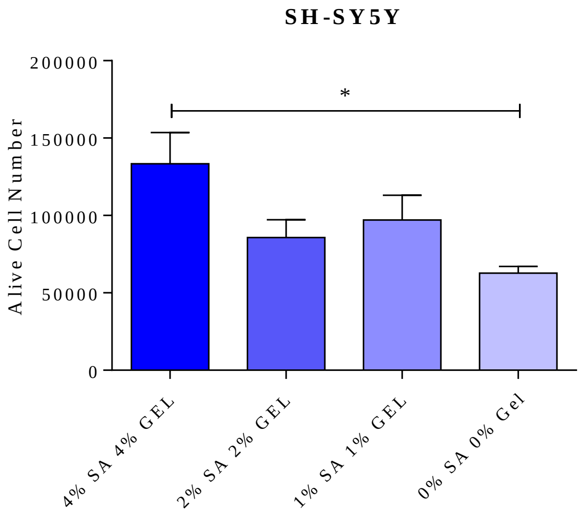

Figure S1. Viability test (LIVE/DEAD® Cell Viability Assays, Invitrogen, USA) for SH-SY5Y cell line after 5 days of culture in the bioink. On the y-axis the number of live cells. 50.000 cells were encapsulated and seeded both in the hydrogel and in the control without bioink. Three concentration of hydrogel were used: 4% SA and 4% GEL, 2% SA and 2% GEL and 1% SA and 1% GEL; 0% SA and 0% GEL indicate the control in 2D environment. Error bars indicates SD. 4% SA and 4% GEL shows a statistically significant increase in the number of live cells compared to 2D control (* $p<0.05$). Data were analyzed by ANOVA ($n = 3$), followed by Newman-Keuls Multiple Comparison Test; * $p < 0.05$.

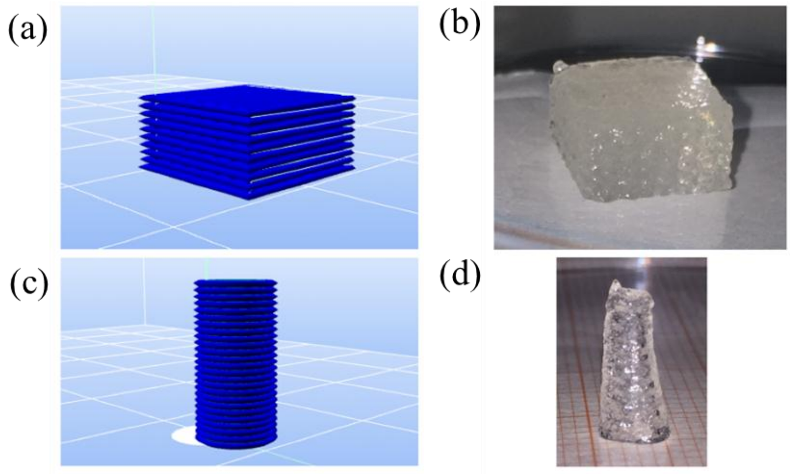

Figure S2. Example of two complex structures: CAD and printed sample using 6% SA and 4% GEL. (a) CAD image of a 10x10 mm cube, composed of 10 layers 0.4 mm each. (b) Resulting printed cube using 6%SA and 4% GEL printed at 25°C setting 70 kPa pressure. (c) CAD image of an empty cylinder composed of 25 layers 0.4 mm each (d) Resulting printed empty cylinder using 6%SA and 4% GEL printed at 25°C setting 55 kPa pressure. Both structures maintained the CAD dimension without collapsing and deforming.

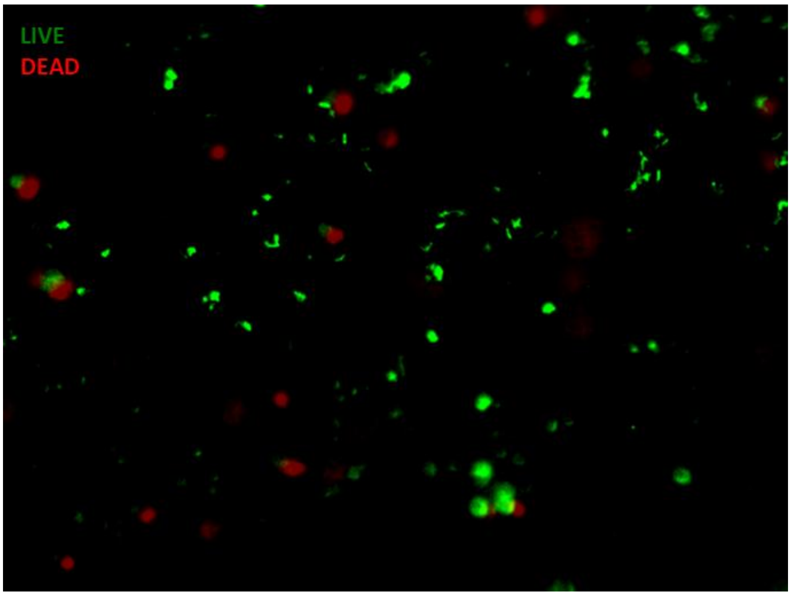

Figure S3. Cellular viability with the LIVE/DEAD® cell viability assay (Invitrogen, USA) in SH-SY5Y encapsulated in 6% SA and 4% GEL after 5 days of culture. Representative Z-stack image of encapsulated SH-SY5Y cells stained with calcein AM (green, live cells) and ethidium homodimer (red, dead cells). Image highlights the presence of live cells (green) compared to dead cells (red) comparable to 50% viability.

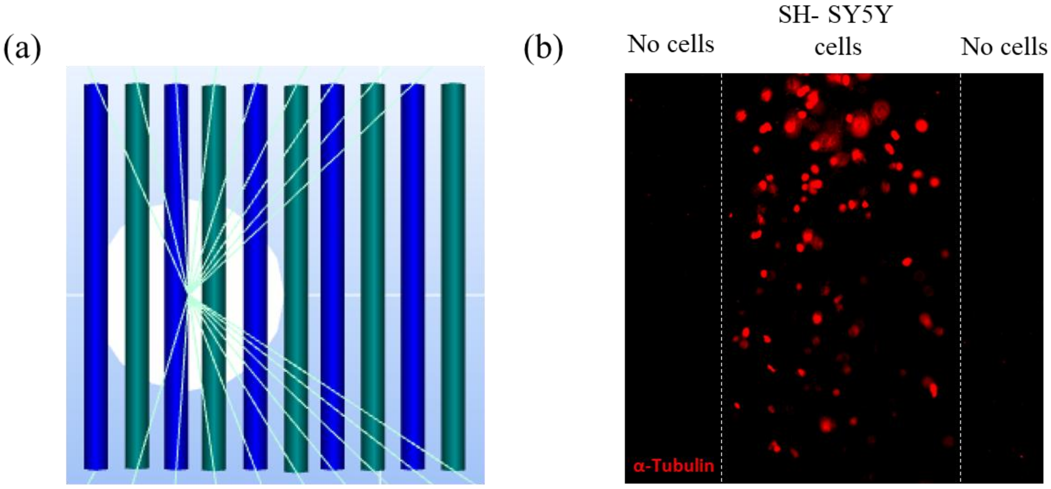

Figure S4. Representation of CAD image and fluorescence image of SH-SY5Y after 5 days of culture. (a) CAD image where blue lines correspond to the bioink without cells and green lines correspond to the bioink with SH-SY5Y cells. (b) Immunofluorescence staining of SH-SY5Y cells after 5 days of culture, marked with α-Tubulin (red), underlines the maintenance of the parallel distribution of cells, facing the lines without cells.
